# Supplementary material for: Soil Layers Impact Lithocarpus Soil Microbial Composition in the Ailao Mountains Subtropical Forest, Yunnan, China
Source: J Fungi (Basel). 2022 Sep 9;8(9):948. doi: 10.3390/jof8090948 (PMC9504396; doi:10.3390/jof8090948)

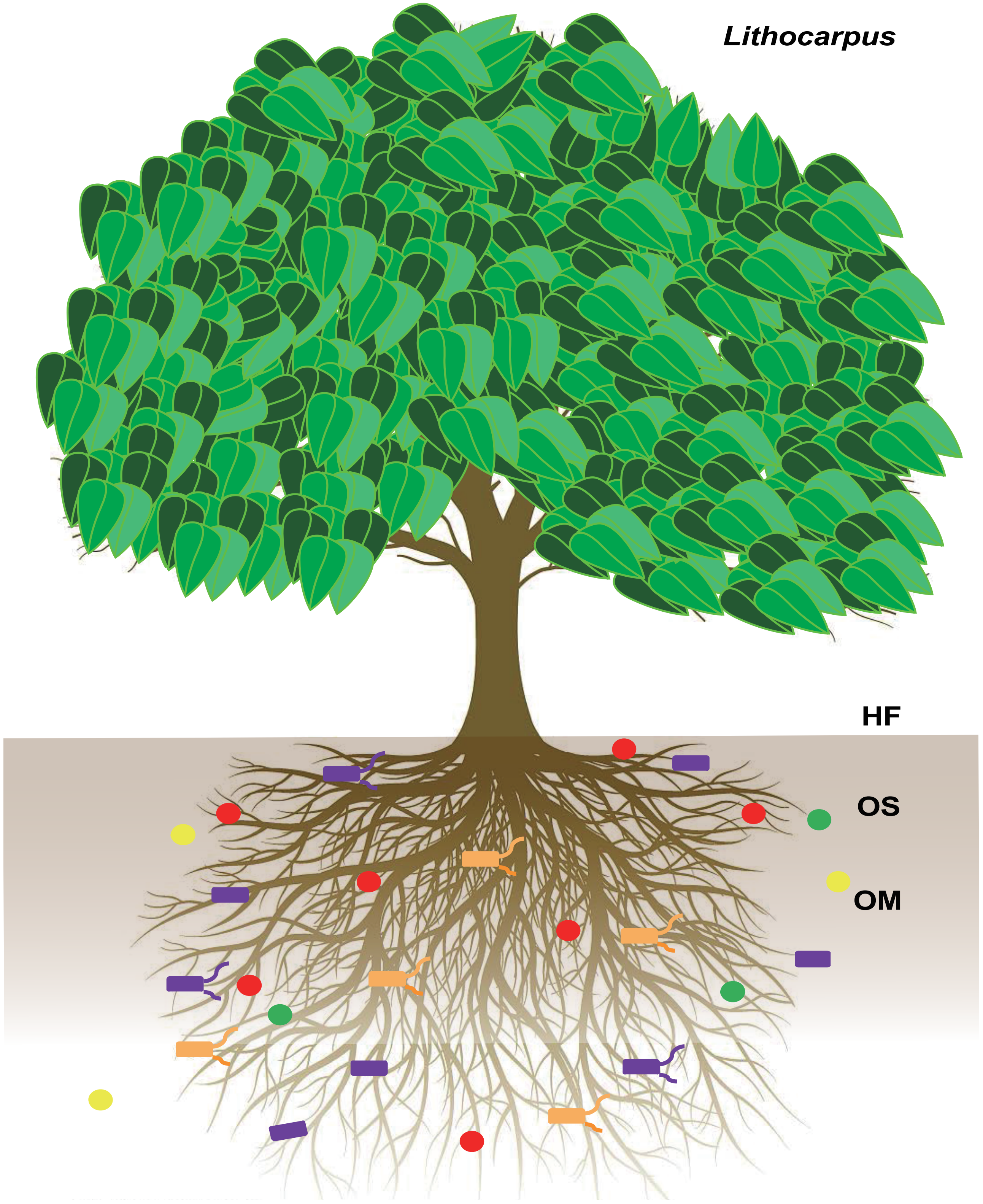

	1. Wet in 2019 (n=72*)		
Different soil layer	Cluster1	Cluster2	Cluster3
HF	T1-T4(n=8)	T5-T8(n=8)	T9-T12(n=8)
OS	T1-T4(n=8)	T5-T8(n=8)	T9-T12(n=8)
OM	T1-T4(n=8)	T5-T8(n=8)	T9-T12(n=8)

(*72=3 soil layer * 2 direction * 4 trees * 3 cluster)

	2. Dry in 2020 (n=72)		
Different soil layer	Cluster1	Cluster2	Cluster3
HF	T1-T4(n=8)	T5-T8(n=8)	T9-T12(n=8)
OS	T1-T4(n=8)	T5-T8(n=8)	T9-T12(n=8)
OM	T1-T4(n=8)	T5-T8(n=8)	T9-T12(n=8)

(*72=3 soil layer * 2 direction * 4 trees * 3 cluster)

	3. Wet in 2020 (n=72)		
Different soil layer	Cluster1	Cluster2	Cluster3
HF	T1-T4(n=8)	T5-T8(n=8)	T9-T12(n=8)
OS	T1-T4(n=8)	T5-T8(n=8)	T9-T12(n=8)
OM	T1-T4(n=8)	T5-T8(n=8)	T9-T12(n=8)

(*72=3 soil layer * 2 direction * 4 trees * 3 cluster)

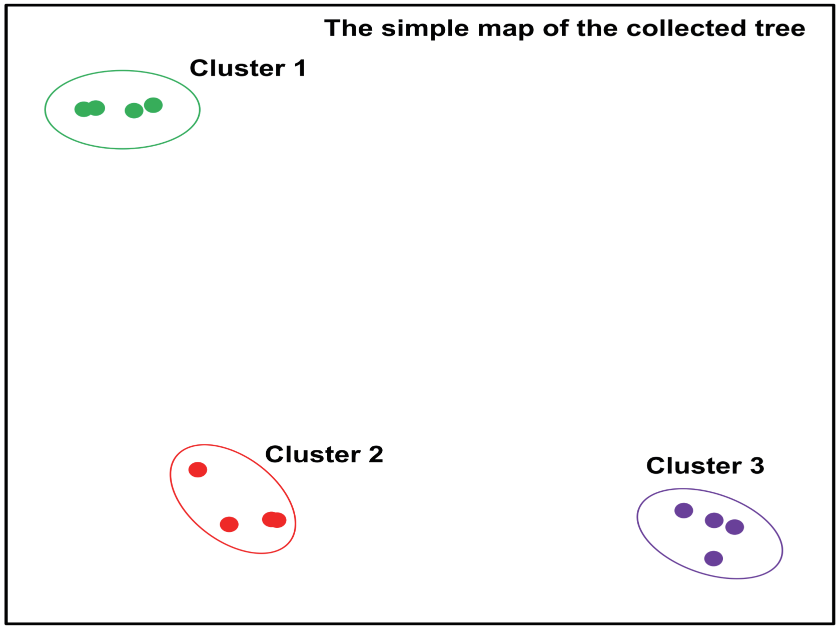

Supplement: Supplementary file 1 [file jof-08-00948-s001.zip › Supplementary materials/Figure S1.pdf]
