# Supplementary material for: Soil Layers Impact Lithocarpus Soil Microbial Composition in the Ailao Mountains Subtropical Forest, Yunnan, China
Source: J Fungi (Basel). 2022 Sep 9;8(9):948. doi: 10.3390/jof8090948 (PMC9504396; doi:10.3390/jof8090948)
Supplement: Supplementary file 1 [file jof-08-00948-s001.zip › Supplementary materials/Figure S11.pdf]

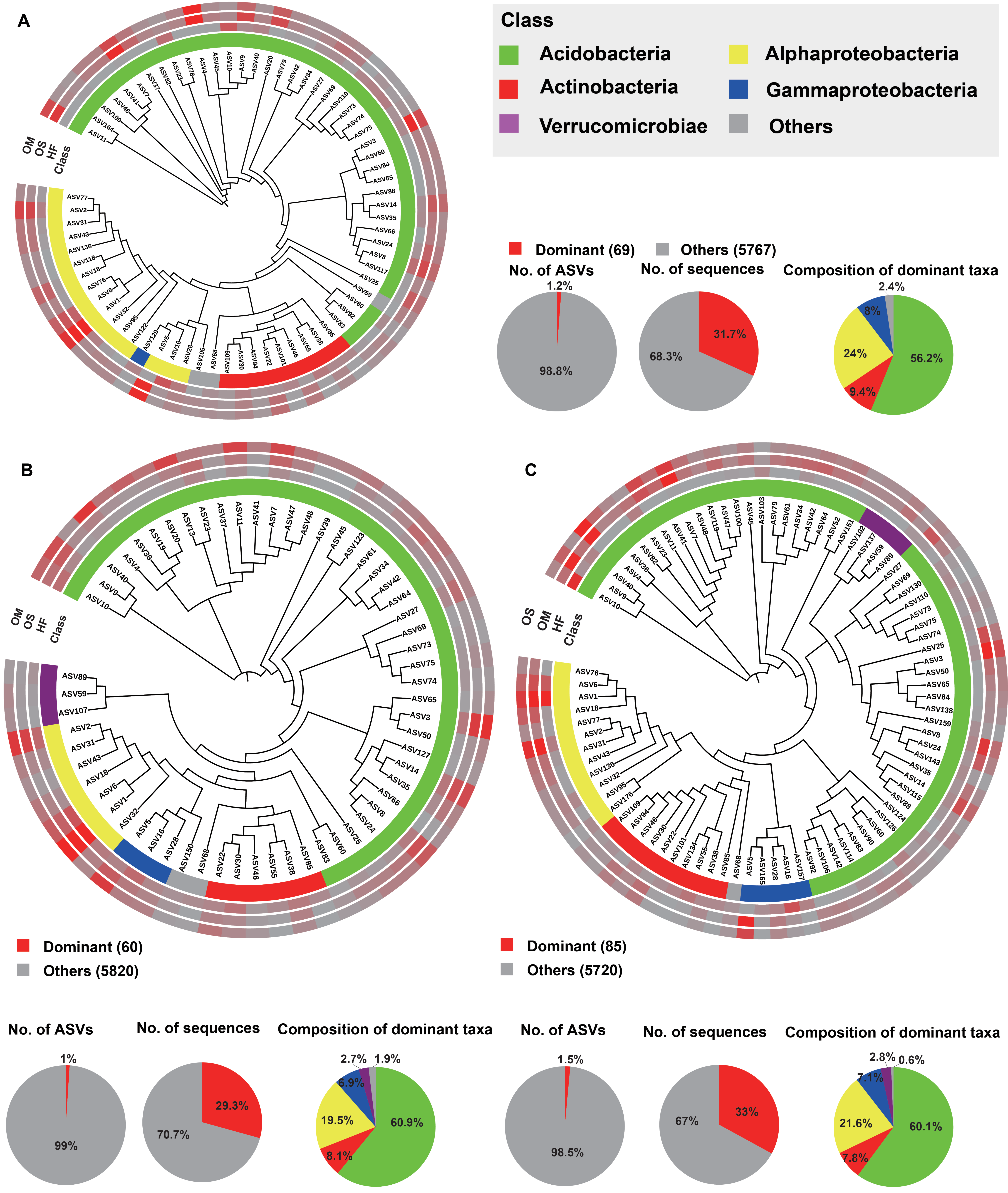

A
B
C
Class
Acidobacteria
Alphaproteobacteria
Actinobacteria
Gammaproteobacteria
Verrucomicrobiae
Others
OM
OS
HF
Class
Dominant (69)
Others (5767)
No. of ASVs
1.2%
98.8%
No. of sequences
31.7%
68.3%
Composition of dominant taxa
2.4%
8%
24%
56.2%
9.4%
Dominant (60)
Others (5820)
No. of ASVs
1%
99%
No. of sequences
29.3%
70.7%
Composition of dominant taxa
2.7%
1.9%
6.9%
19.5%
60.9%
8.1%
Dominant (85)
Others (5720)
No. of ASVs
1.5%
98.5%
No. of sequences
33%
67%
Composition of dominant taxa
2.8%
0.6%
7.1%
21.6%
60.1%
7.8%
