# Supplementary material for: Soil Layers Impact Lithocarpus Soil Microbial Composition in the Ailao Mountains Subtropical Forest, Yunnan, China
Source: J Fungi (Basel). 2022 Sep 9;8(9):948. doi: 10.3390/jof8090948 (PMC9504396; doi:10.3390/jof8090948)
Supplement: Supplementary file 1 [file jof-08-00948-s001.zip › Supplementary materials/Figure S12.pdf]

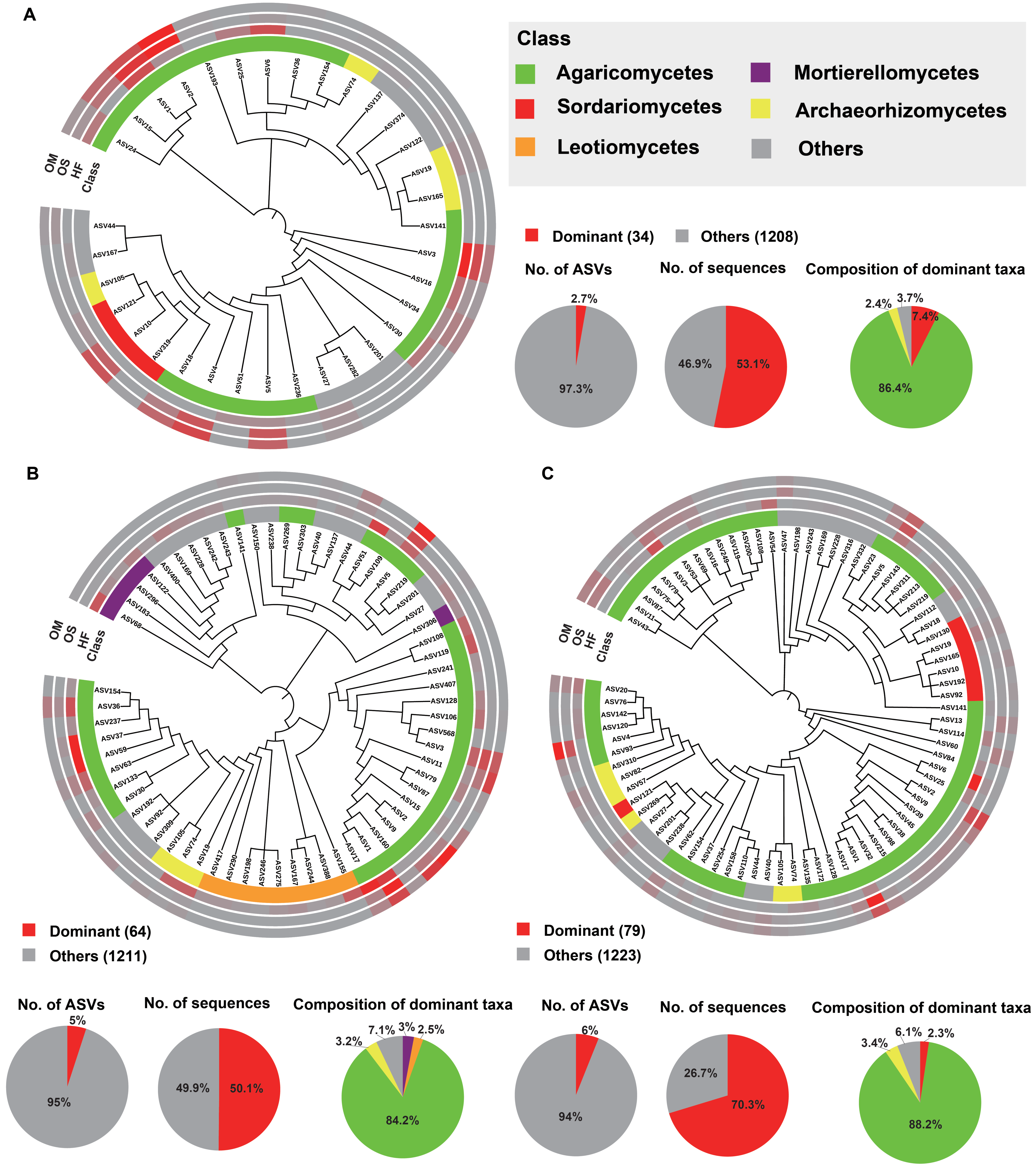
A
Class
Agaricomycetes
Sordariomycetes
Leotiomycetes
Mortierellomycetes
Archaeorhizomycetes
Others
OM
OS
HF
Class
Dominant (34)
Others (1208)
No. of ASVs
2.7%
97.3%
No. of sequences
46.9%
53.1%
Composition of dominant taxa
2.4%
3.7%
7.4%
86.4%
B
OM
OS
HF
Class
Dominant (64)
Others (1211)
No. of ASVs
5%
95%
No. of sequences
49.9%
50.1%
Composition of dominant taxa
3.2%
7.1%
3%
2.5%
84.2%
C
OM
OS
HF
Class
Dominant (79)
Others (1223)
No. of ASVs
6%
94%
No. of sequences
26.7%
70.3%
Composition of dominant taxa
3.4%
6.1%
2.3%
88.2%
