# Supplementary material for: Soil Layers Impact Lithocarpus Soil Microbial Composition in the Ailao Mountains Subtropical Forest, Yunnan, China
Source: J Fungi (Basel). 2022 Sep 9;8(9):948. doi: 10.3390/jof8090948 (PMC9504396; doi:10.3390/jof8090948)
Supplement: Supplementary file 1 [file jof-08-00948-s001.zip › Supplementary materials/Figure S3.pdf]

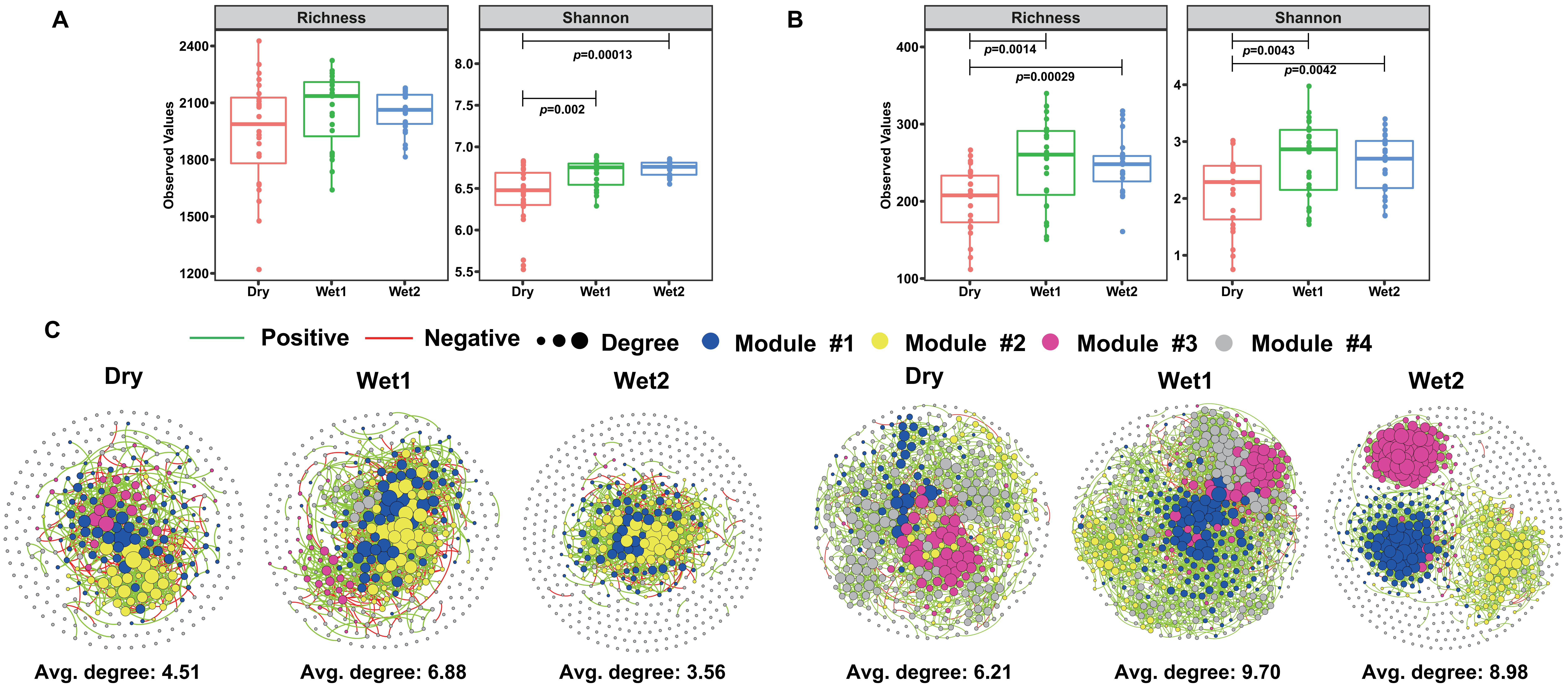
A
Richness
Shannon
Observed Values
2400
2100
1800
1500
1200
8.0
7.5
7.0
6.5
6.0
5.5
p=0.00013
p=0.002
Dry
Wet1
Wet2
B
Richness
Shannon
400
300
200
100
4
3
2
1
p=0.0014
p=0.00029
p=0.0043
p=0.0042
C
Positive
Negative
Degree
Module #1
Module #2
Module #3
Module #4
Avg. degree: 4.51
Avg. degree: 6.88
Avg. degree: 3.56
Avg. degree: 6.21
Avg. degree: 9.70
Avg. degree: 8.98
