# Supplementary material for: Soil Layers Impact Lithocarpus Soil Microbial Composition in the Ailao Mountains Subtropical Forest, Yunnan, China
Source: J Fungi (Basel). 2022 Sep 9;8(9):948. doi: 10.3390/jof8090948 (PMC9504396; doi:10.3390/jof8090948)
Supplement: Supplementary file 1 [file jof-08-00948-s001.zip › Supplementary materials/Figure S6.pdf]

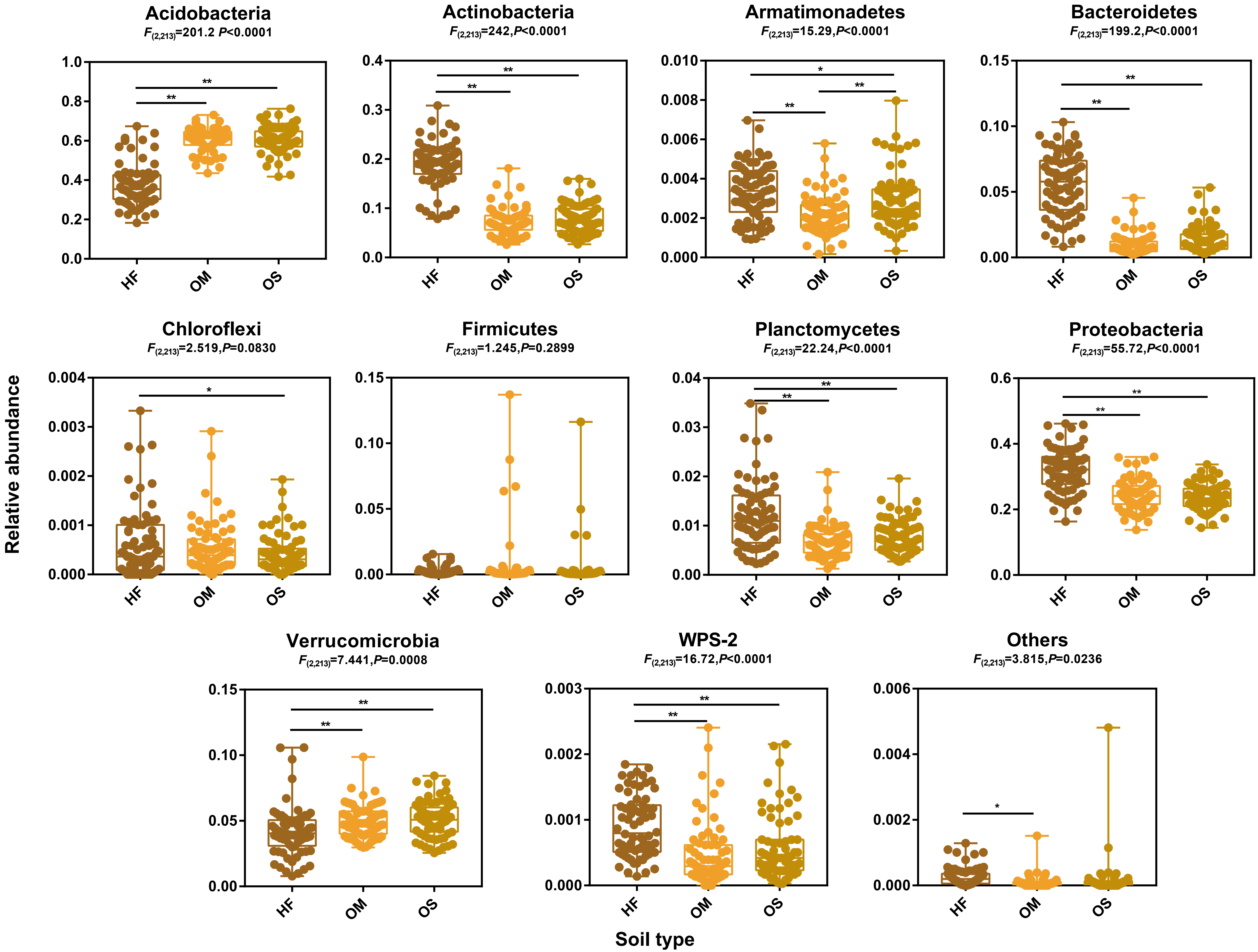

Acidobacteria
$F_{(2,213)}$=201.2 P<0.0001
Actinobacteria
$F_{(2,213)}$=242, P<0.0001
Armatimonadetes
$F_{(2,213)}$=15.29, P<0.0001
Bacteroidetes
$F_{(2,213)}$=199.2, P<0.0001
Chloroflexi
$F_{(2,213)}$=2.519, P=0.0830
Firmicutes
$F_{(2,213)}$=1.245, P=0.2899
Planctomycetes
$F_{(2,213)}$=22.24, P<0.0001
Proteobacteria
$F_{(2,213)}$=55.72, P<0.0001
Verrucomicrobia
$F_{(2,213)}$=7.441, P=0.0008
WPS-2
$F_{(2,213)}$=16.72, P<0.0001
Others
$F_{(2,213)}$=3.815, P=0.0236
HF
OM
OS
Relative abundance
Soil type
