# Supplementary material for: Soil Layers Impact Lithocarpus Soil Microbial Composition in the Ailao Mountains Subtropical Forest, Yunnan, China
Source: J Fungi (Basel). 2022 Sep 9;8(9):948. doi: 10.3390/jof8090948 (PMC9504396; doi:10.3390/jof8090948)
Supplement: Supplementary file 1 [file jof-08-00948-s001.zip › Supplementary materials/Figure S7.pdf]

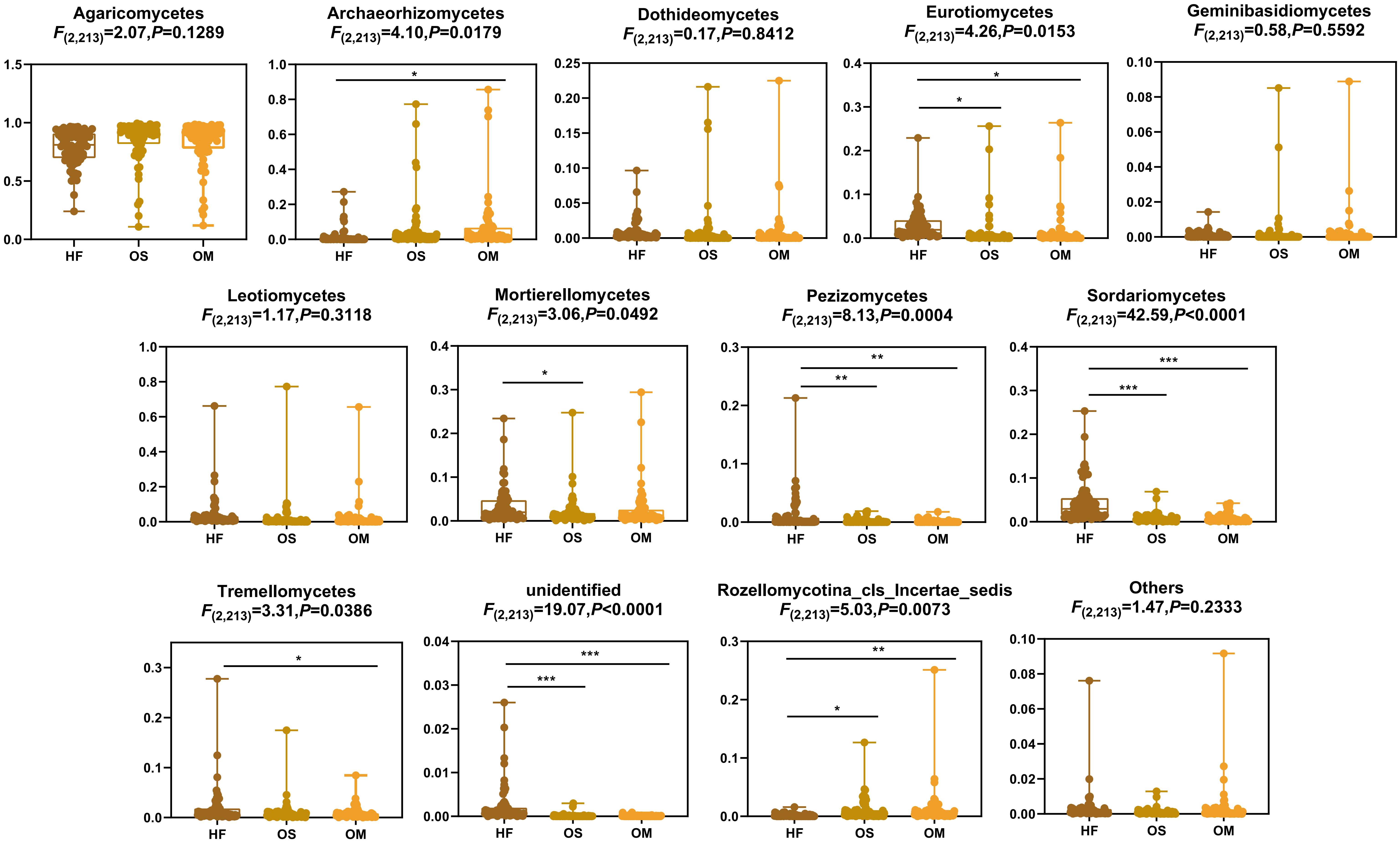

Agaricomycetes
F(2,213)=2.07,P=0.1289
Archaeorhizomycetes
F(2,213)=4.10,P=0.0179
Dothideomycetes
F(2,213)=0.17,P=0.8412
Eurotiomycetes
F(2,213)=4.26,P=0.0153
Geminibasidiomycetes
F(2,213)=0.58,P=0.5592
Leotiomycetes
F(2,213)=1.17,P=0.3118
Mortierellomycetes
F(2,213)=3.06,P=0.0492
Pezizomycetes
F(2,213)=8.13,P=0.0004
Sordariomycetes
F(2,213)=42.59,P<0.0001
Tremellomycetes
F(2,213)=3.31,P=0.0386
unidentified
F(2,213)=19.07,P<0.0001
Rozellomycotina_cls_Incertae_sedis
F(2,213)=5.03,P=0.0073
Others
F(2,213)=1.47,P=0.2333
HF
OS
OM
