# Supplementary material for: Soil Layers Impact Lithocarpus Soil Microbial Composition in the Ailao Mountains Subtropical Forest, Yunnan, China
Source: J Fungi (Basel). 2022 Sep 9;8(9):948. doi: 10.3390/jof8090948 (PMC9504396; doi:10.3390/jof8090948)
Supplement: Supplementary file 1 [file jof-08-00948-s001.zip › Supplementary materials/Figure S8.pdf]

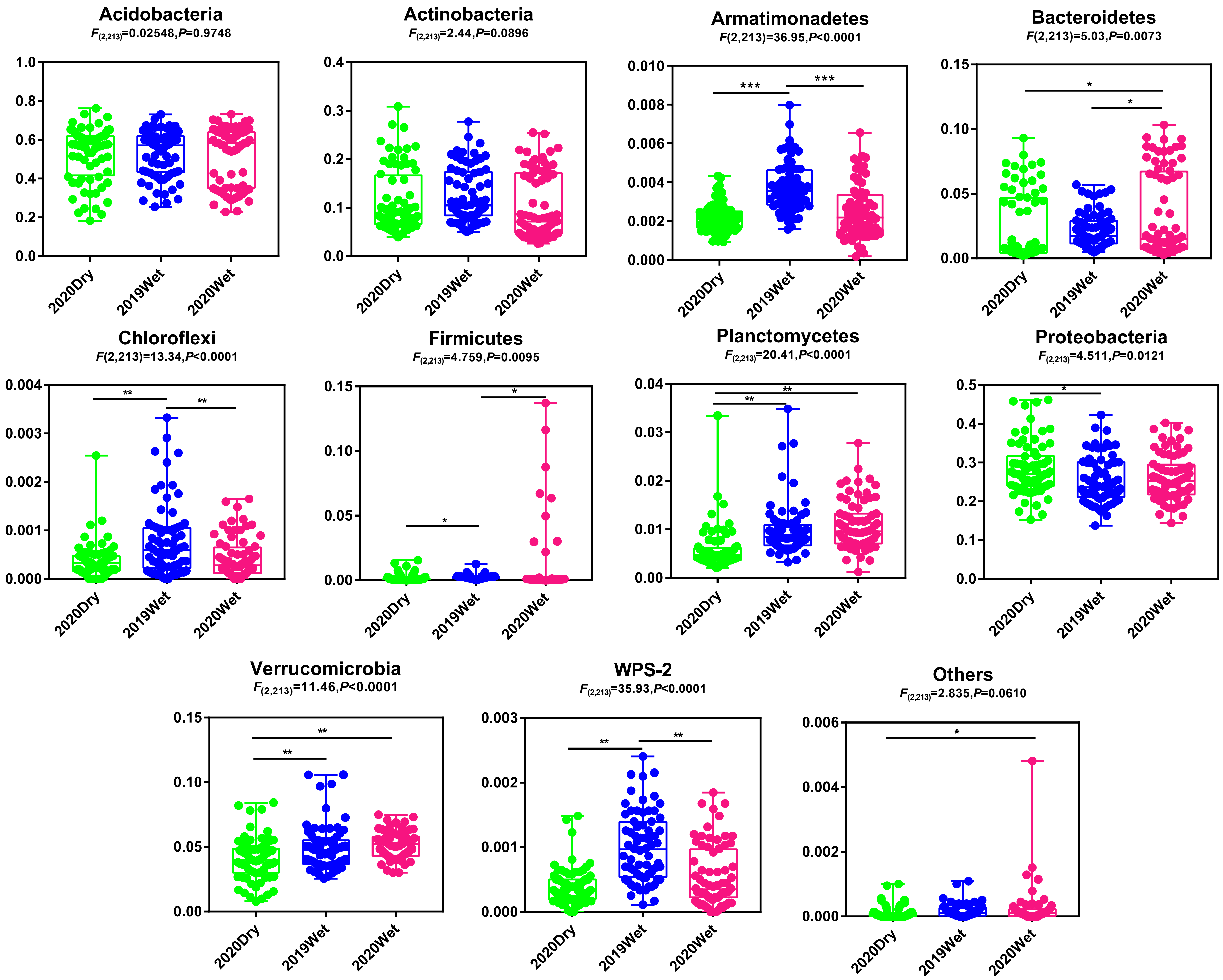

Acidobacteria
F(2,213)=0.02548,P=0.9748
Actinobacteria
F(2,213)=2.44,P=0.0896
Armatimonadetes
F(2,213)=36.95,P<0.0001
Bacteroidetes
F(2,213)=5.03,P=0.0073
Chloroflexi
F(2,213)=13.34,P<0.0001
Firmicutes
F(2,213)=4.759,P=0.0095
Planctomycetes
F(2,213)=20.41,P<0.0001
Proteobacteria
F(2,213)=4.511,P=0.0121
Verrucomicrobia
F(2,213)=11.46,P<0.0001
WPS-2
F(2,213)=35.93,P<0.0001
Others
F(2,213)=2.835,P=0.0610
2020Dry
2019Wet
2020Wet
