# Supplementary material for: Soil Layers Impact Lithocarpus Soil Microbial Composition in the Ailao Mountains Subtropical Forest, Yunnan, China
Source: J Fungi (Basel). 2022 Sep 9;8(9):948. doi: 10.3390/jof8090948 (PMC9504396; doi:10.3390/jof8090948)
Supplement: Supplementary file 1 [file jof-08-00948-s001.zip › Supplementary materials/Figure S9.pdf]

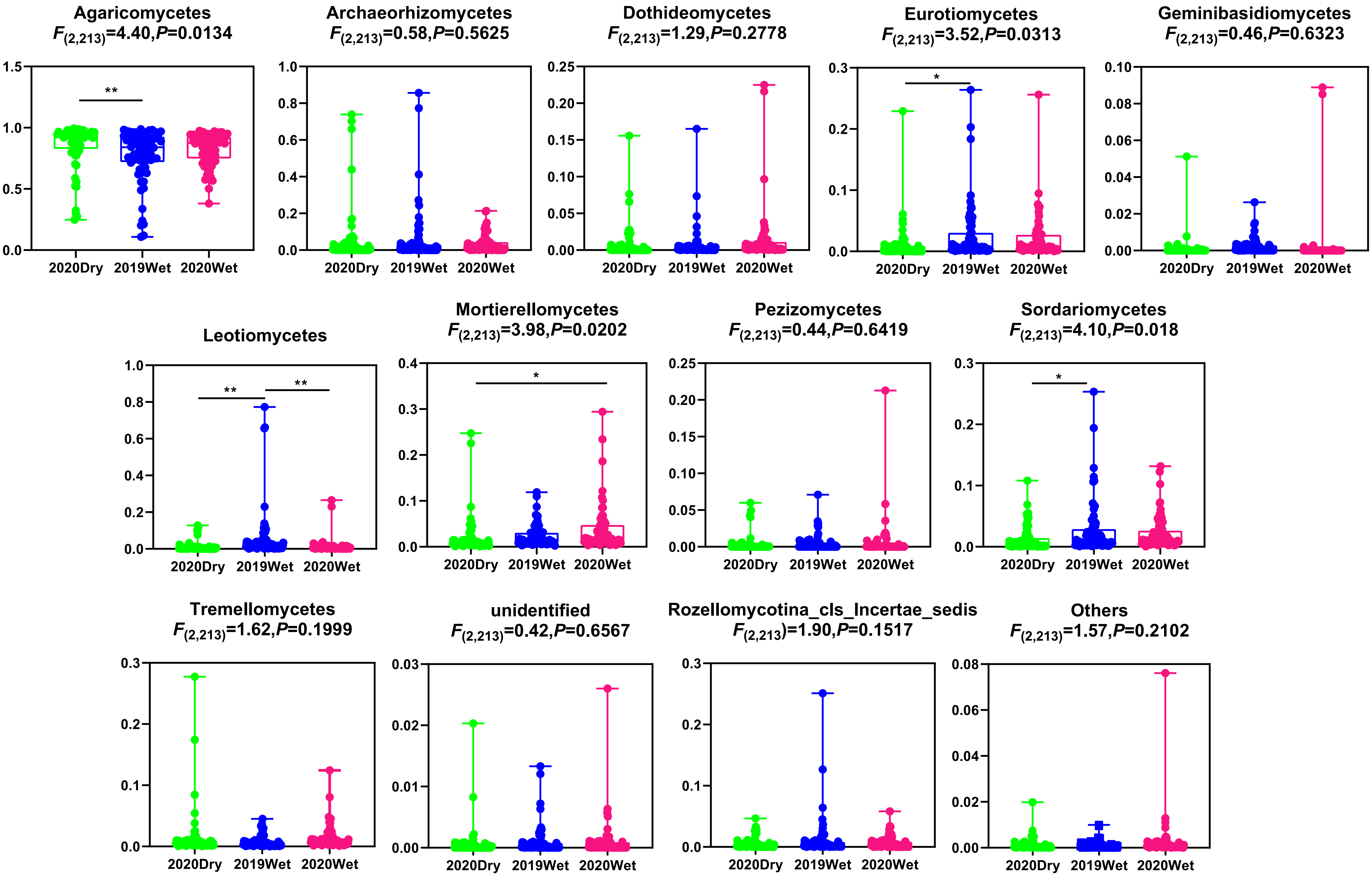

Agaricomycetes
$F_{(2,213)}$=4.40,P=0.0134
Archaeorhizomycetes
$F_{(2,213)}$=0.58,P=0.5625
Dothideomycetes
$F_{(2,213)}$=1.29,P=0.2778
Eurotiomycetes
$F_{(2,213)}$=3.52,P=0.0313
Geminibasidiomycetes
$F_{(2,213)}$=0.46,P=0.6323
Leotiomycetes
Mortierellomycetes
$F_{(2,213)}$=3.98,P=0.0202
Pezizomycetes
$F_{(2,213)}$=0.44,P=0.6419
Sordariomycetes
$F_{(2,213)}$=4.10,P=0.018
Tremellomycetes
$F_{(2,213)}$=1.62,P=0.1999
unidentified
$F_{(2,213)}$=0.42,P=0.6567
Rozellomycotina_cls_Incertae_sedis
$F_{(2,213)}$=1.90,P=0.1517
Others
$F_{(2,213)}$=1.57,P=0.2102
2020Dry
2019Wet
2020Wet
