# Supplementary figures and images for: Soil Layers Impact Lithocarpus Soil Microbial Composition in the Ailao Mountains Subtropical Forest, Yunnan, China
Source: J Fungi (Basel). 2022 Sep 9;8(9):948. doi: 10.3390/jof8090948 (PMC9504396; doi:10.3390/jof8090948)

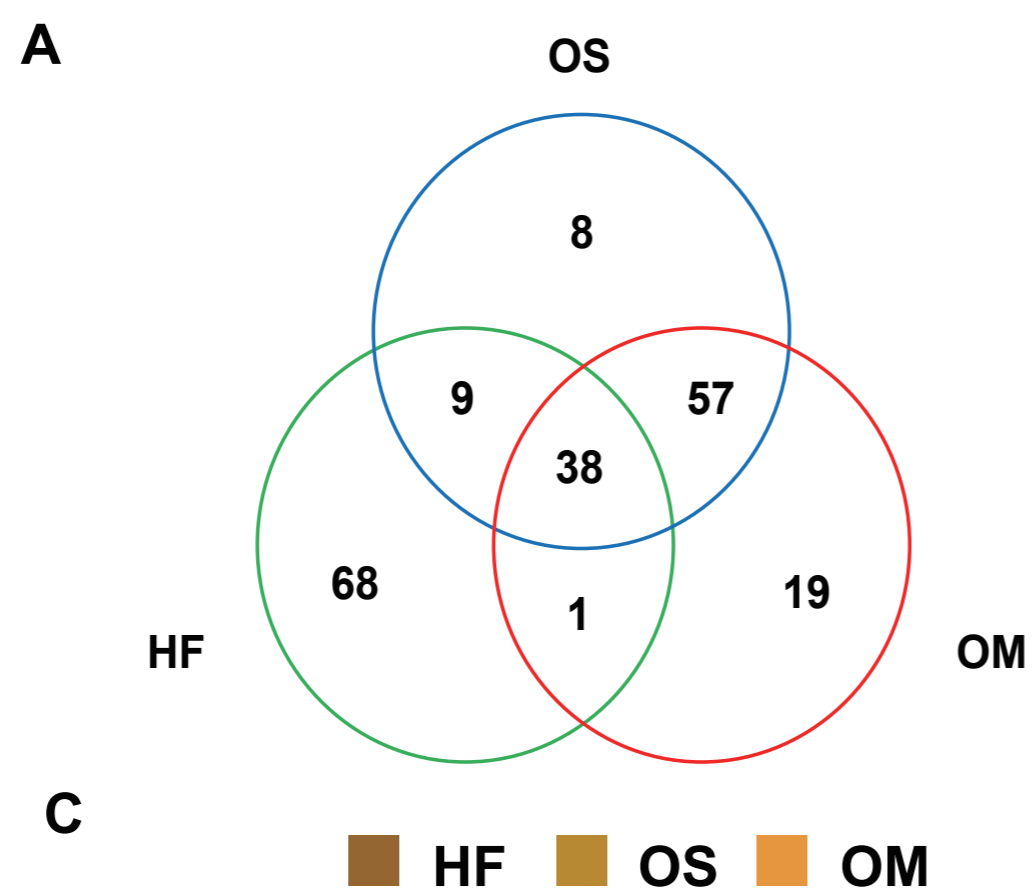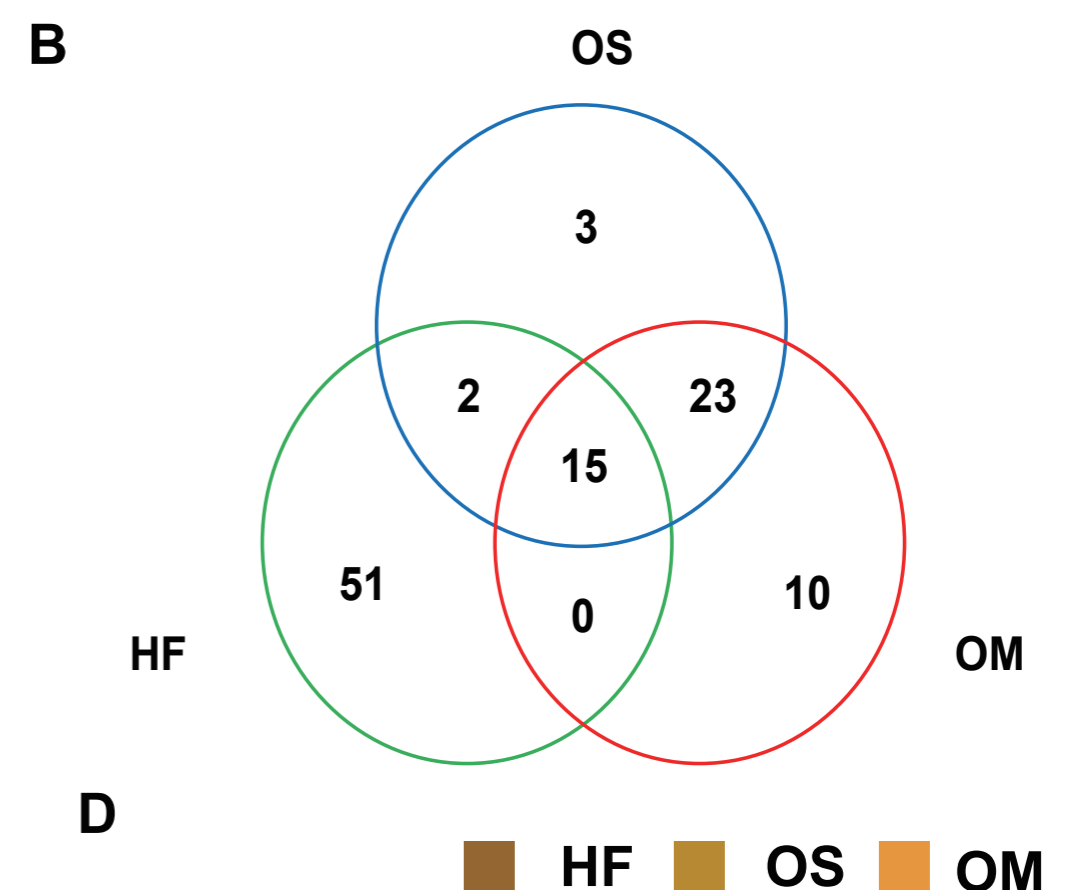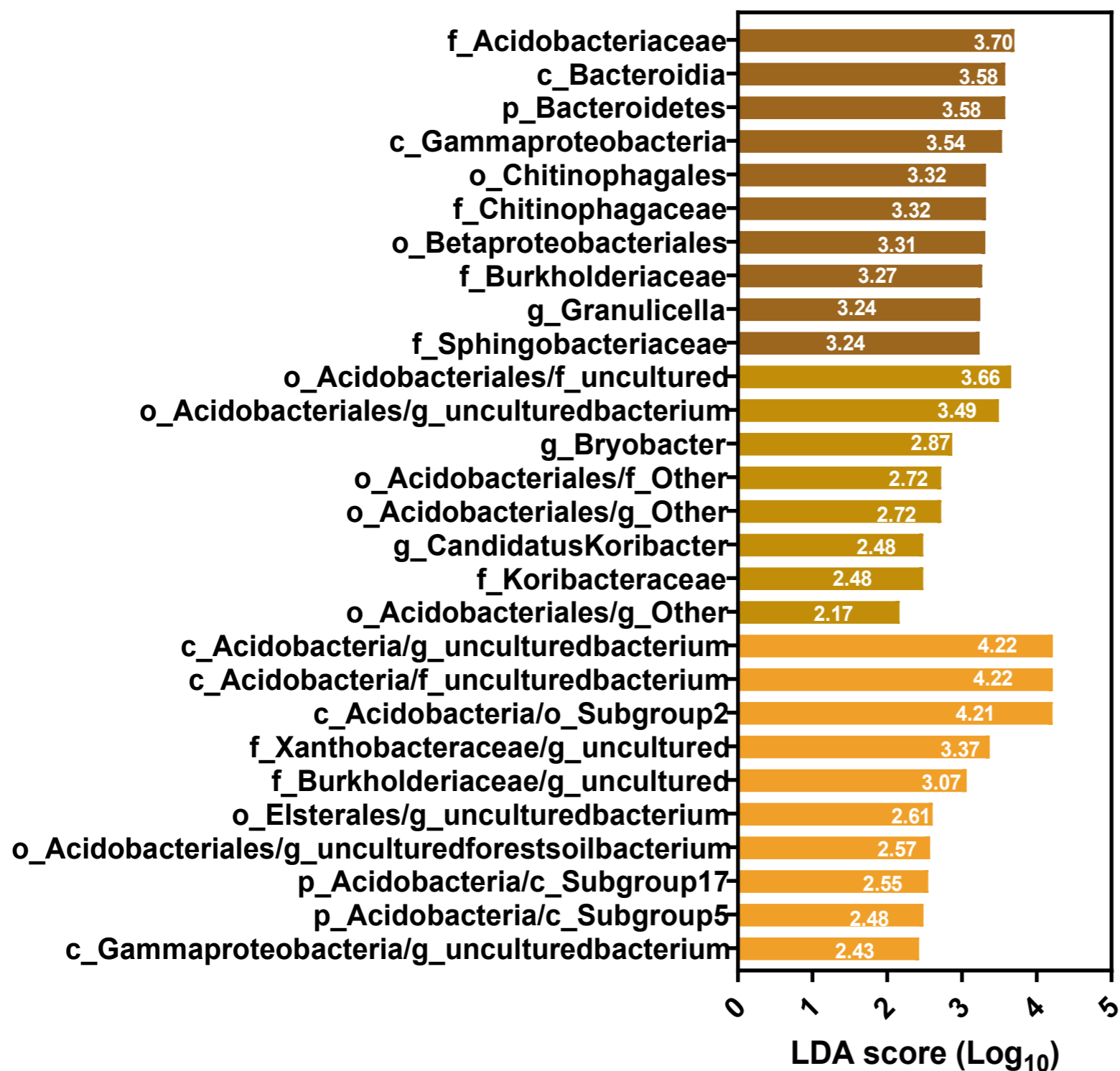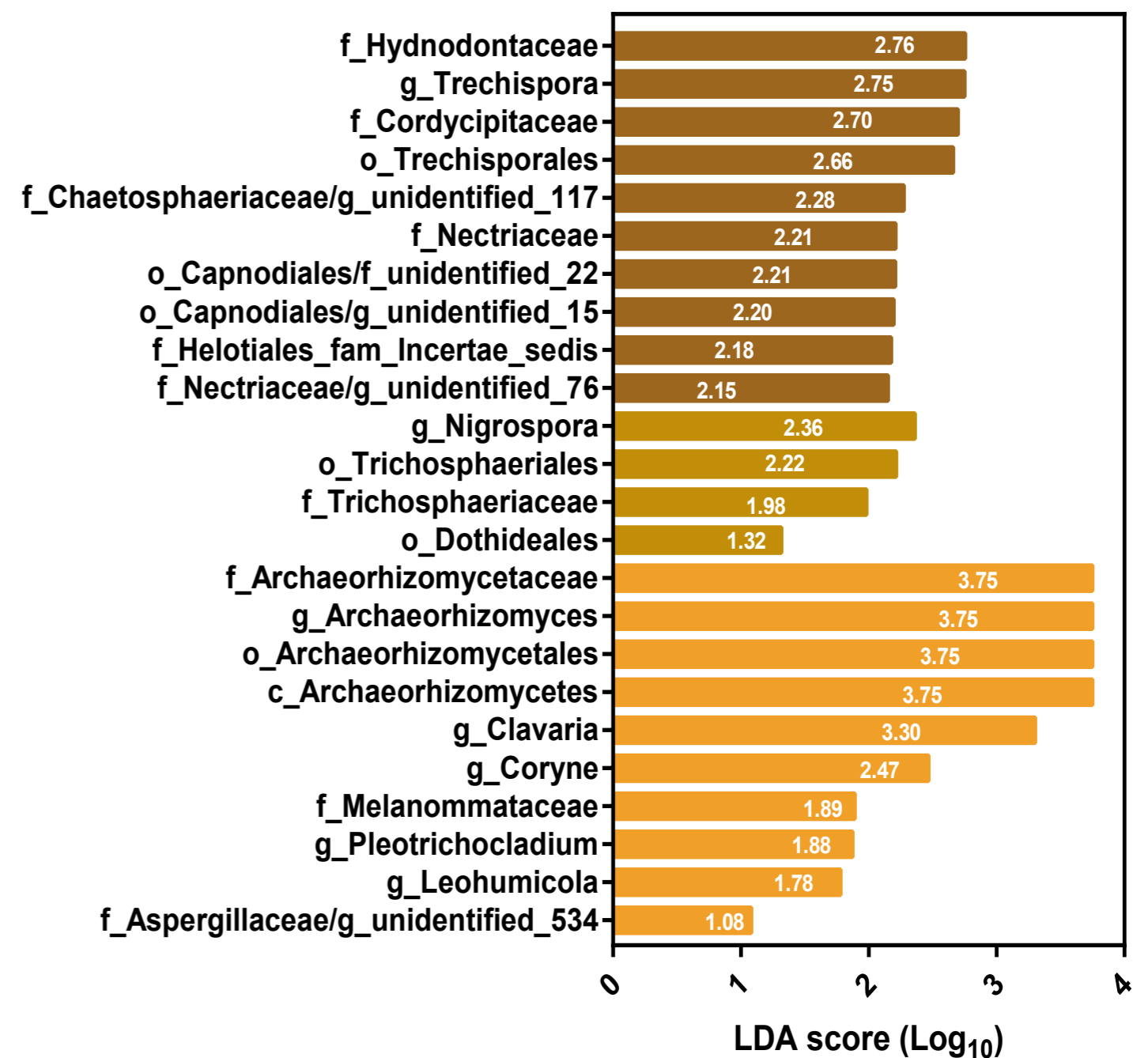

Supplement: Supplementary file 1 [file jof-08-00948-s001.zip › Supplementary materials/Figure S10.pdf]

A

2020Wet

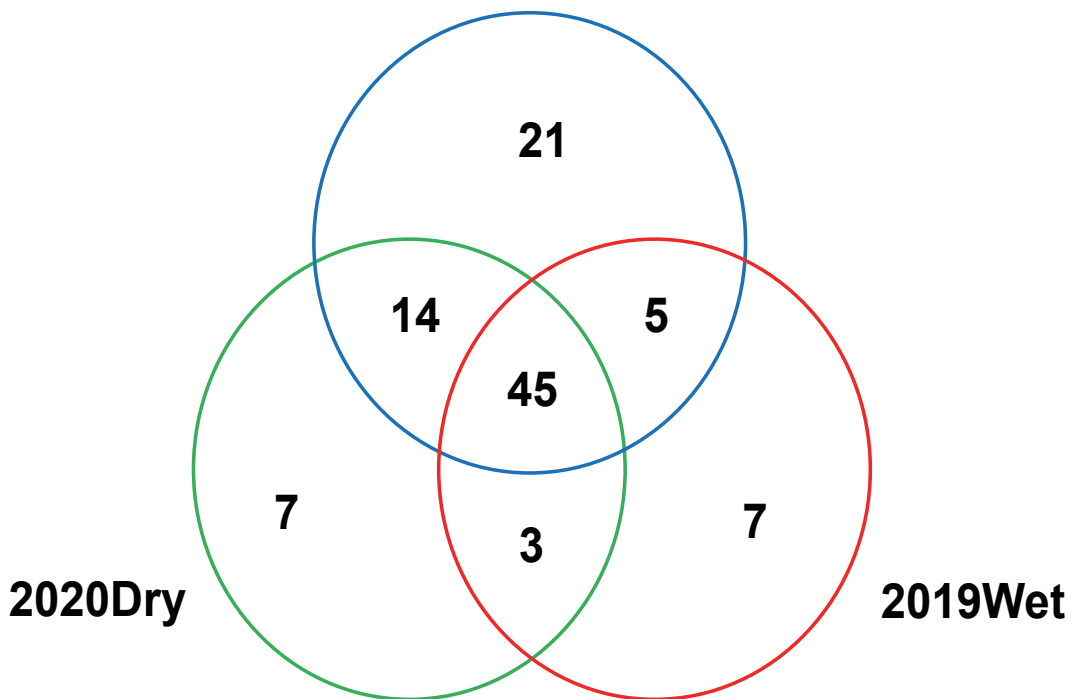

C

2020Dry 2019Wet 2020Wet

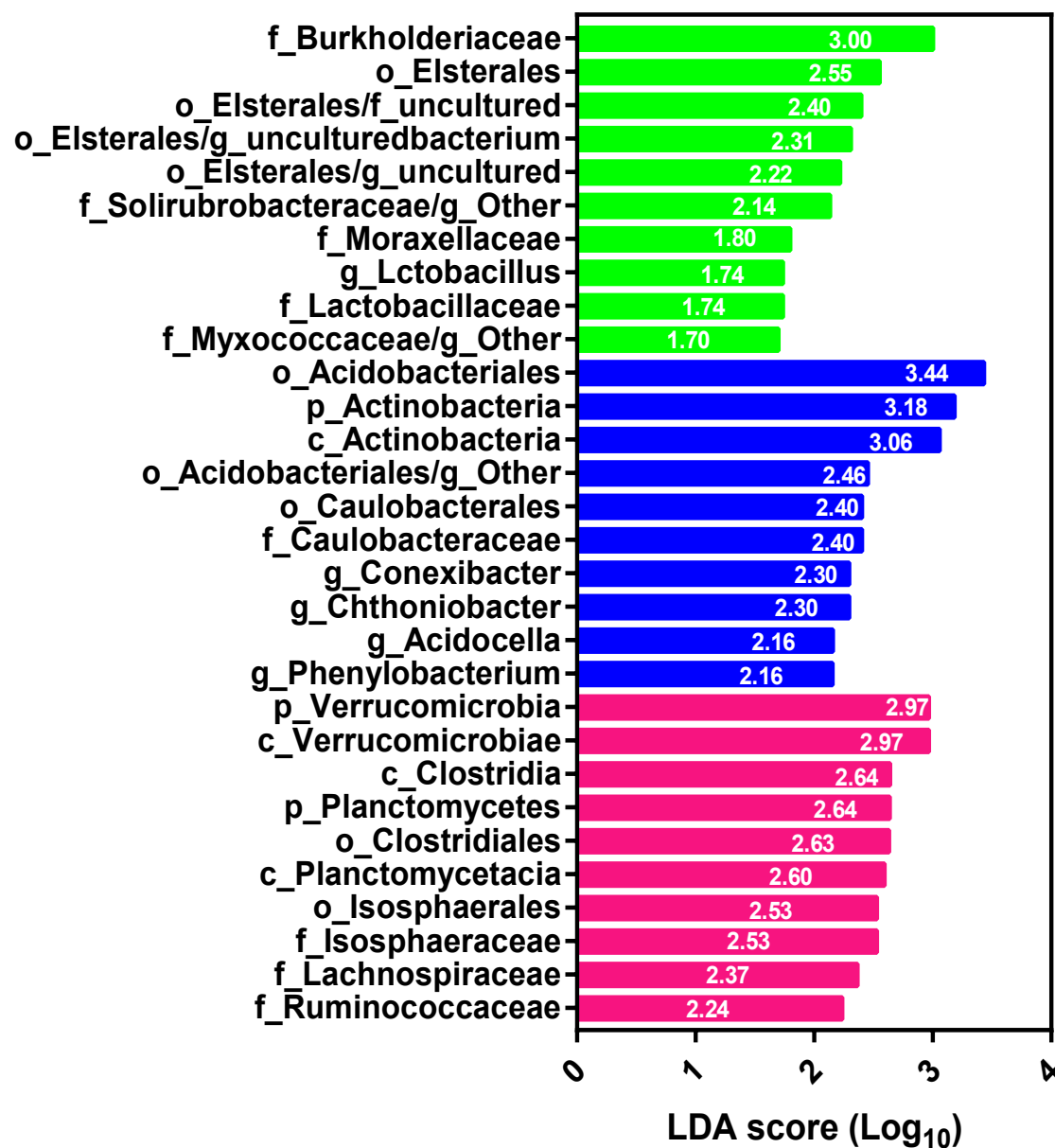

B

2020Wet

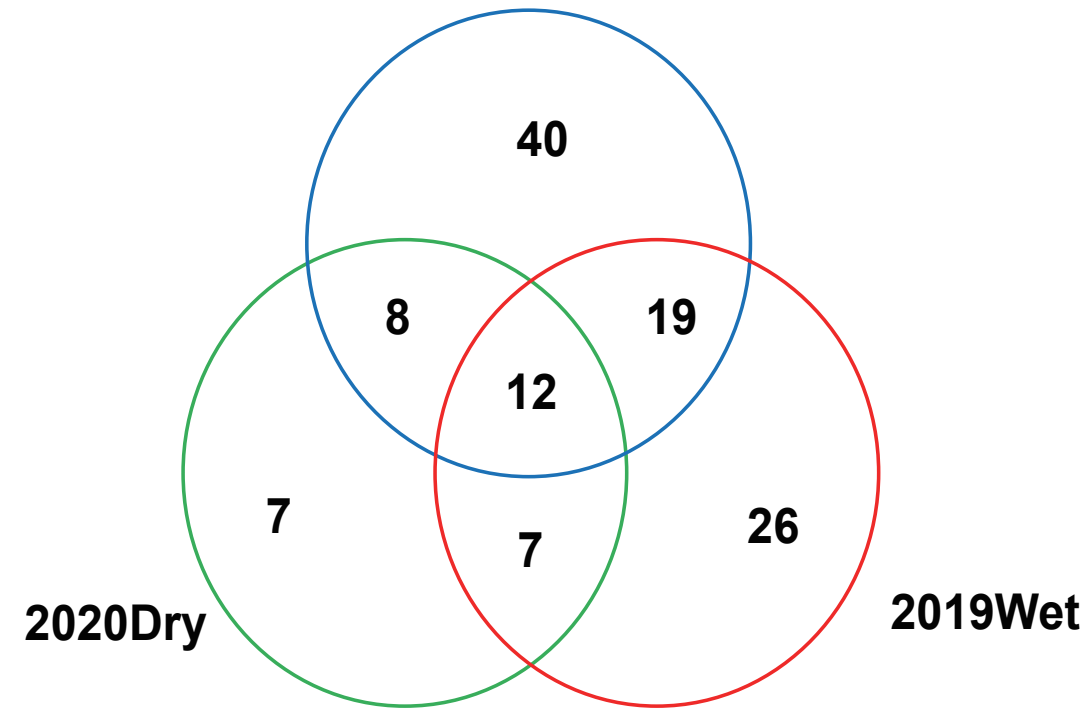

D

2020Dry 2019Wet 2020Wet

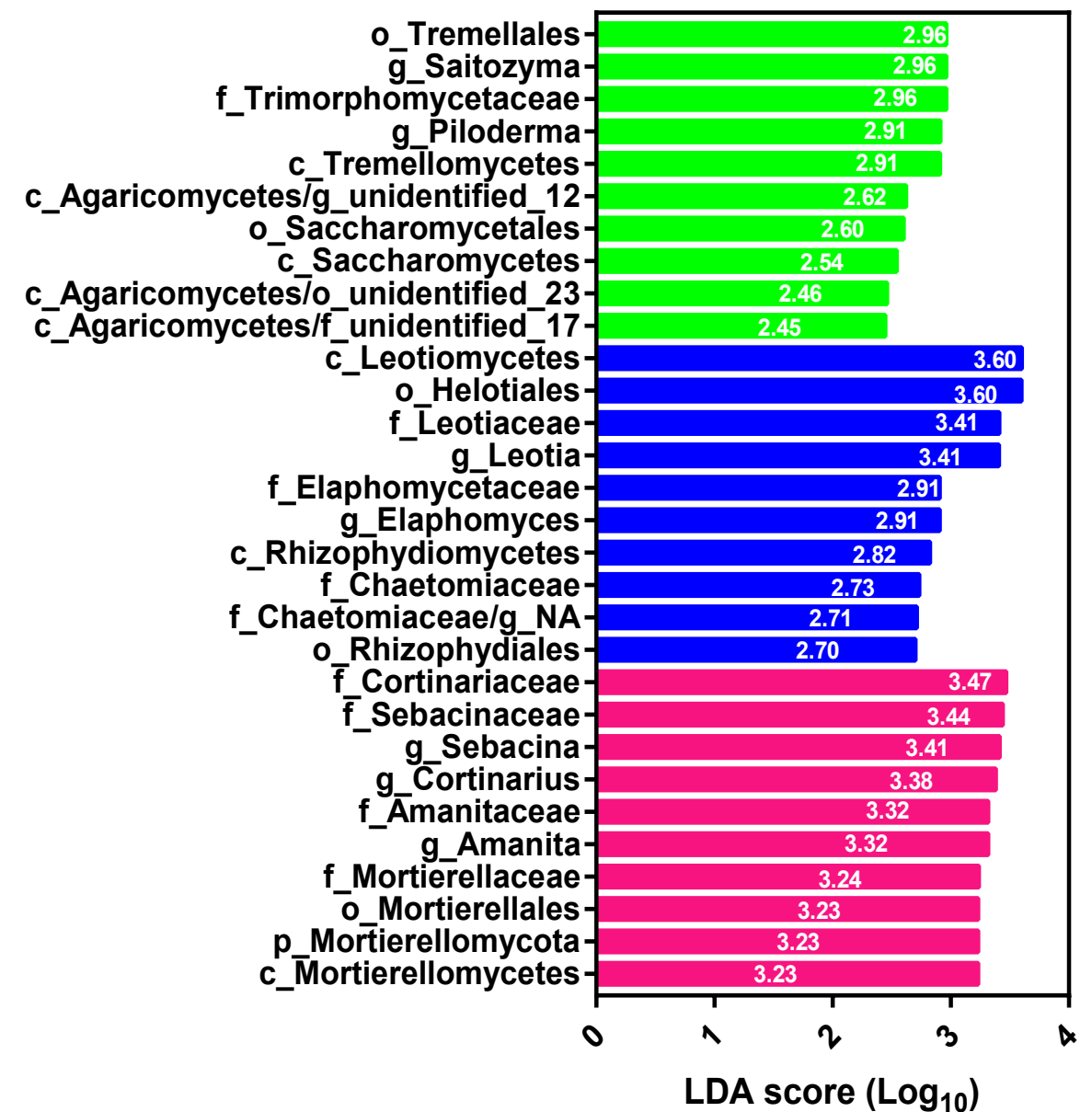

Supplement: Supplementary file 1 [file jof-08-00948-s001.zip › Supplementary materials/Figure S13.pdf]

A

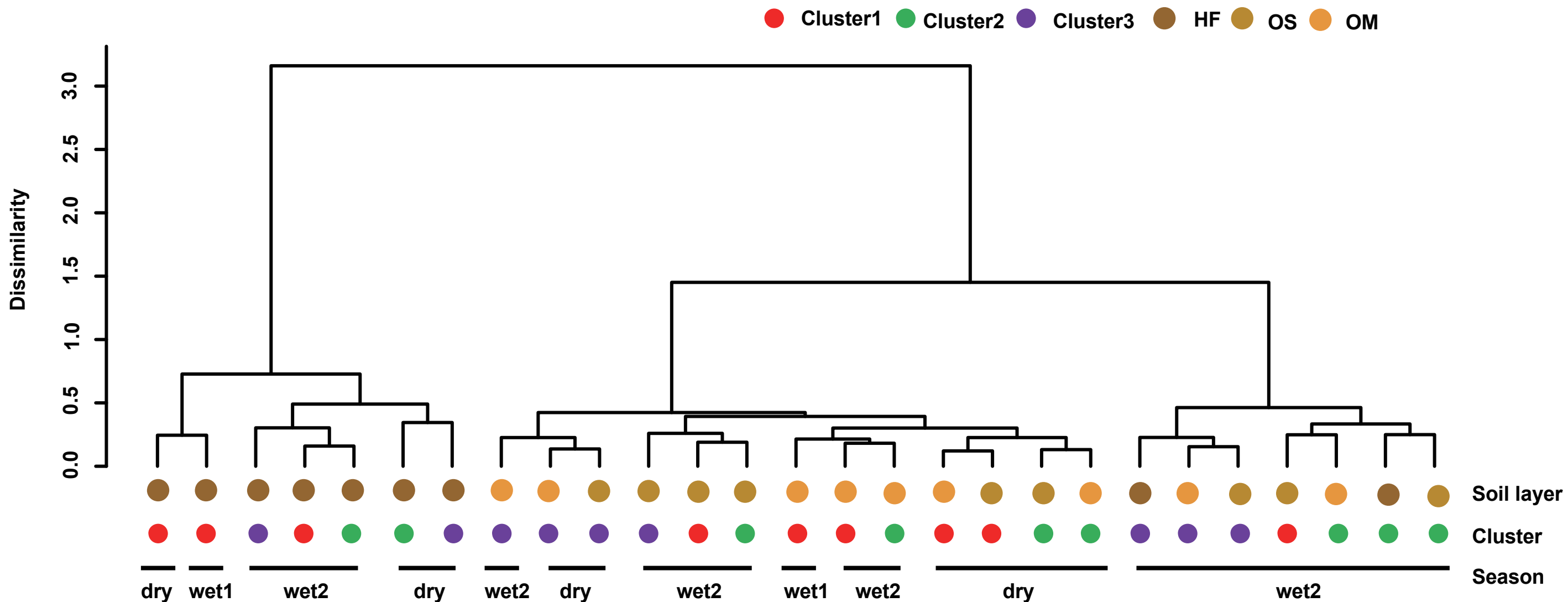

B

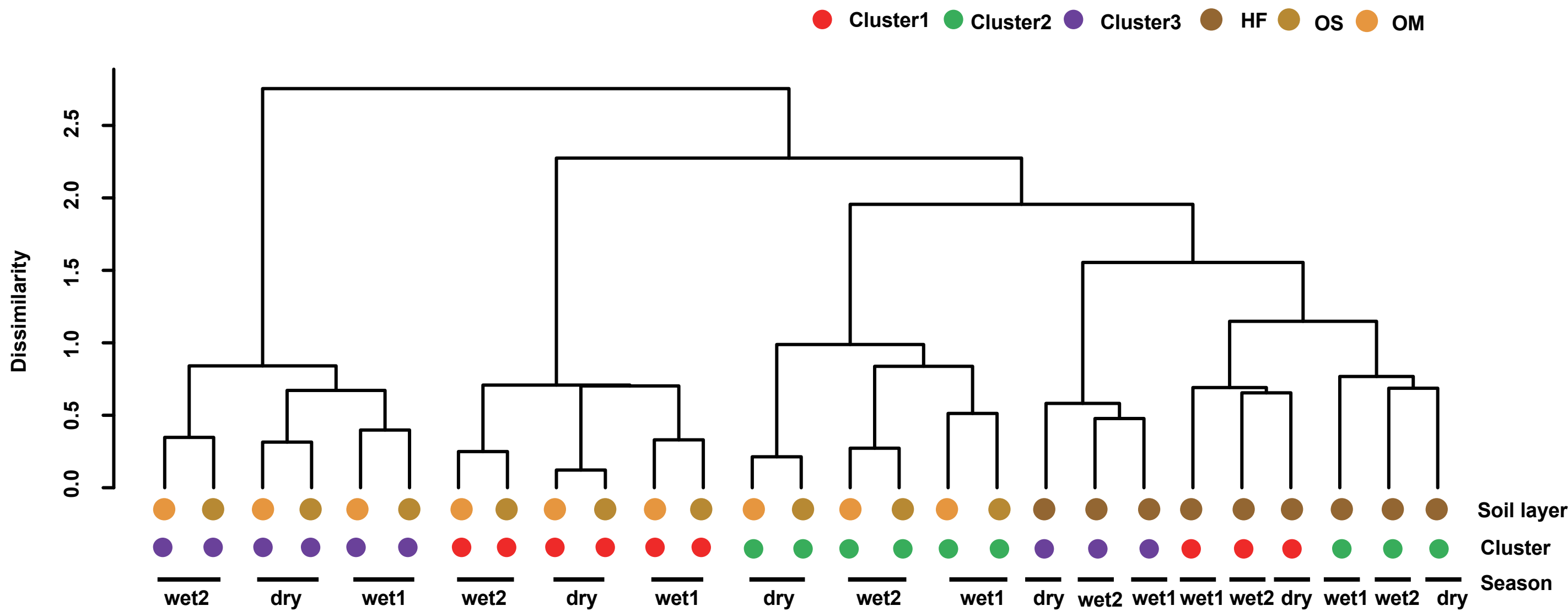

Supplement: Supplementary file 1 [file jof-08-00948-s001.zip › Supplementary materials/Figure S2.pdf]

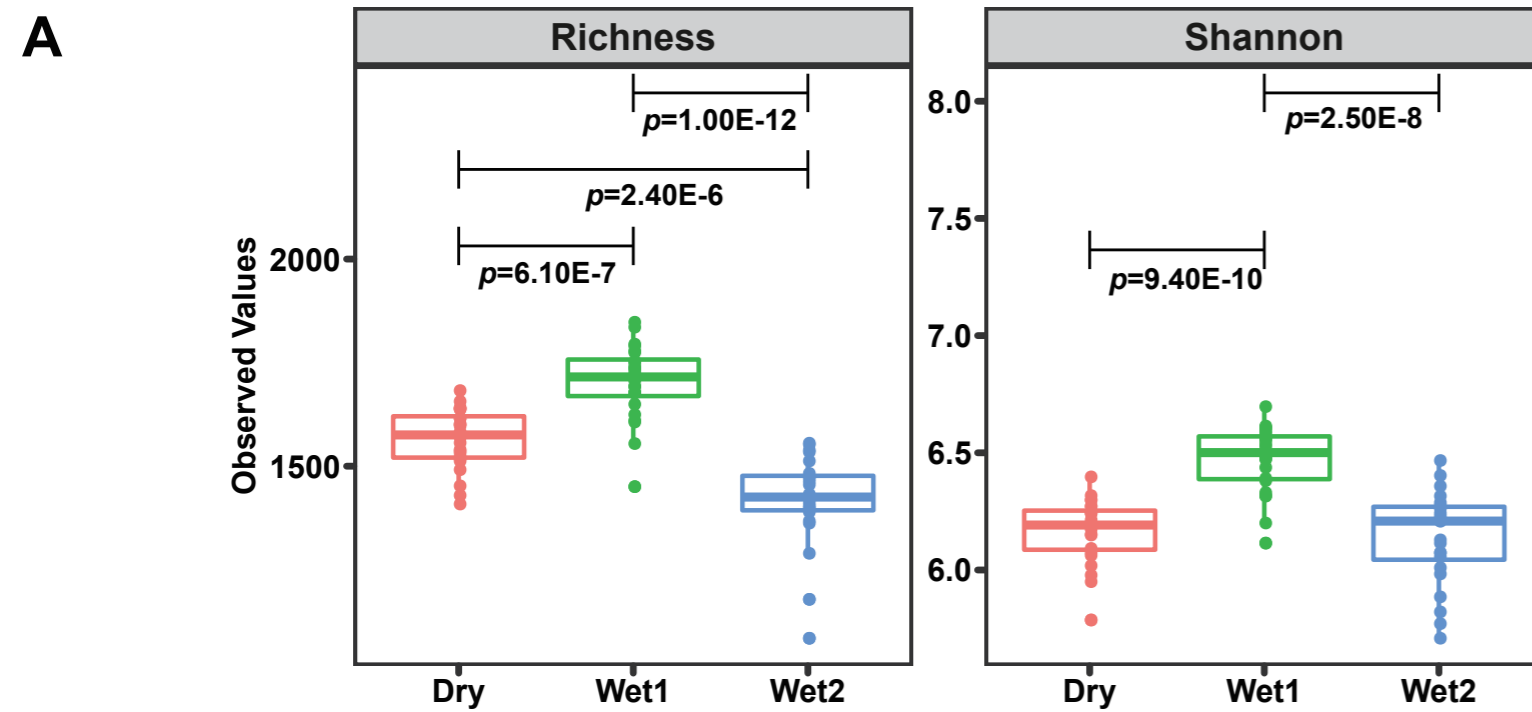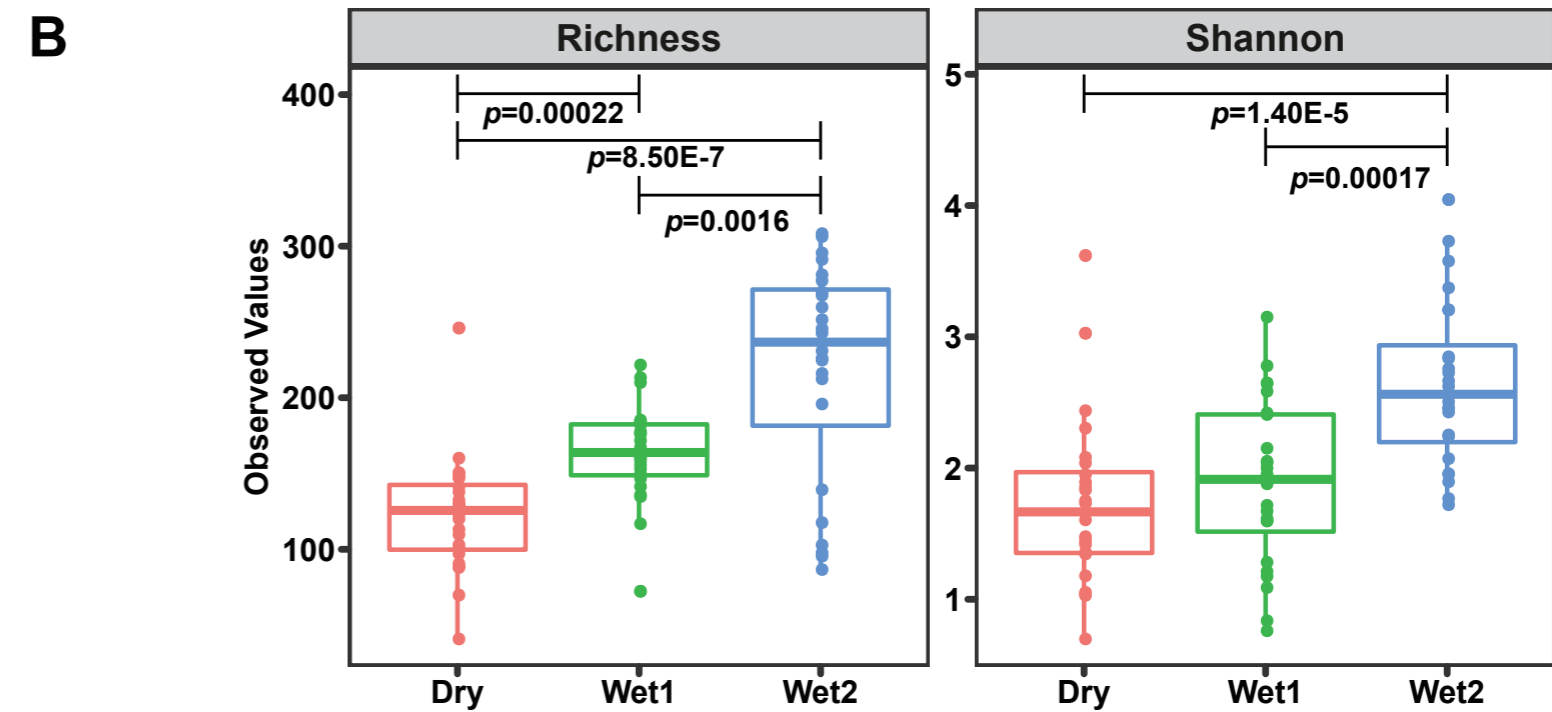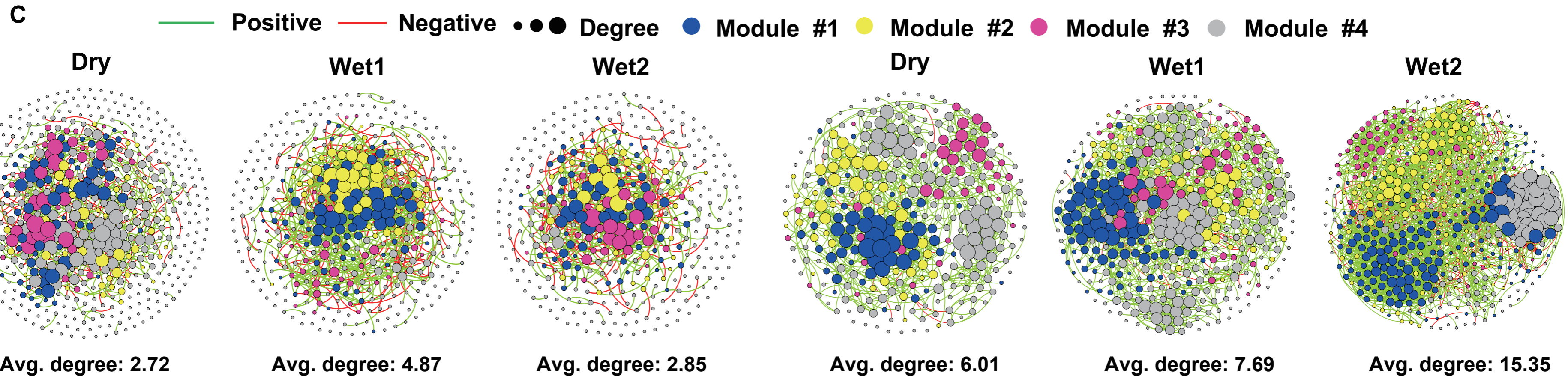

Supplement: Supplementary file 1 [file jof-08-00948-s001.zip › Supplementary materials/Figure S4.pdf]

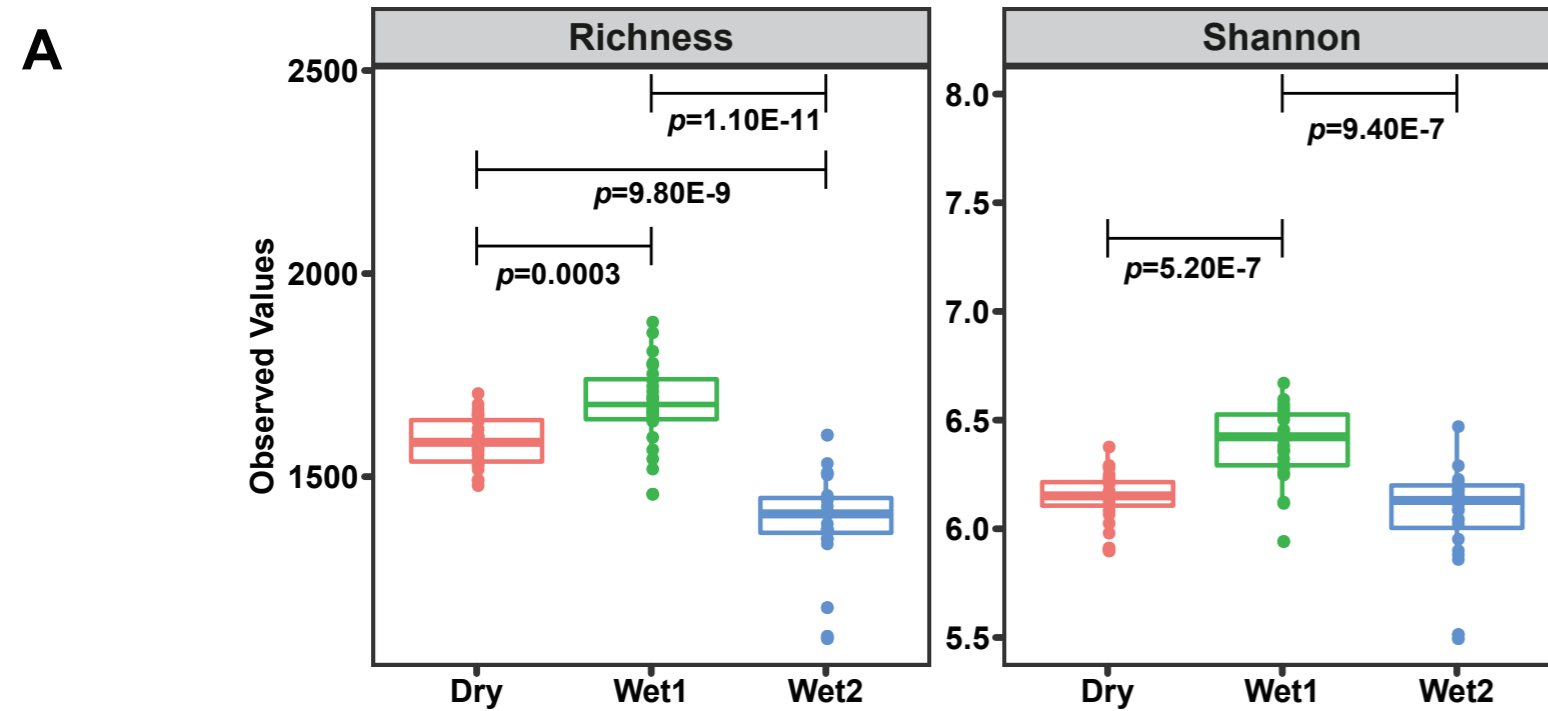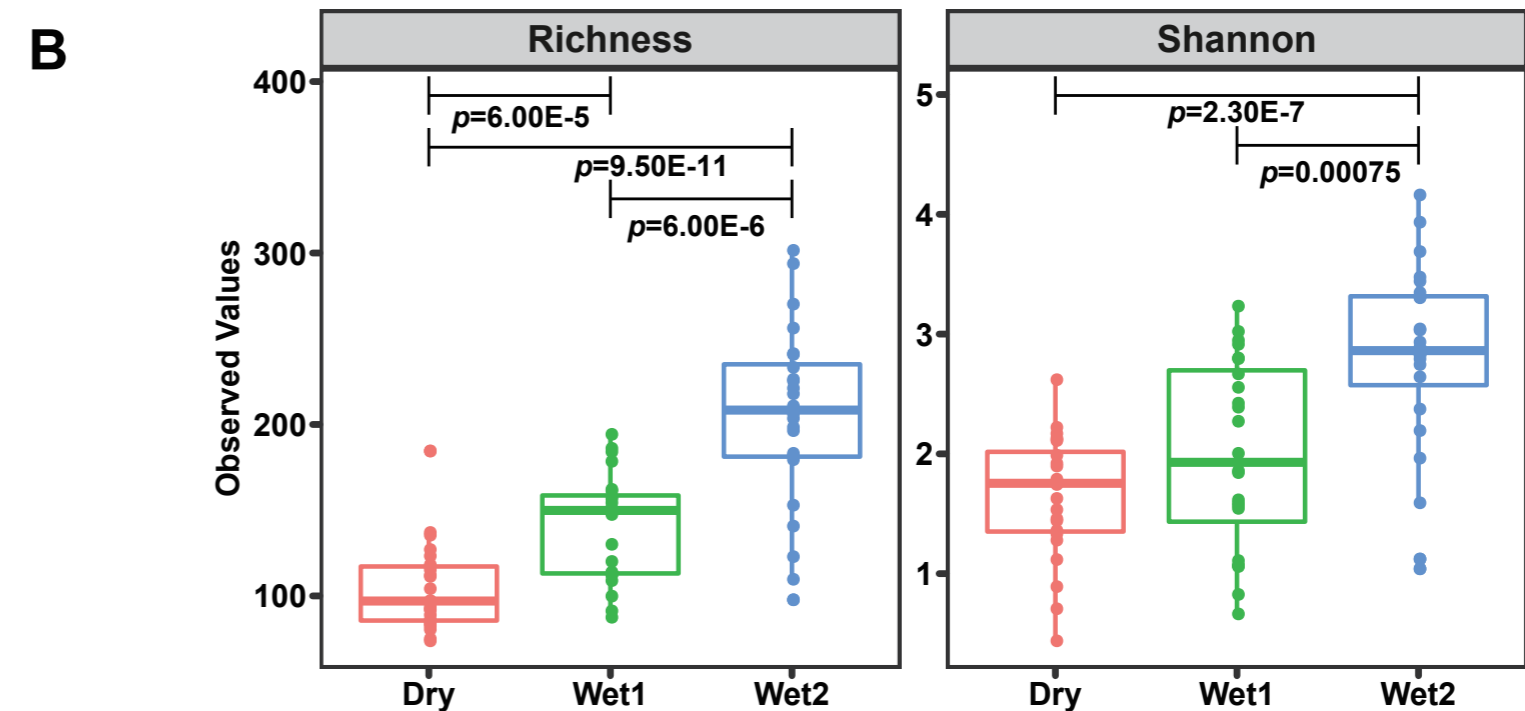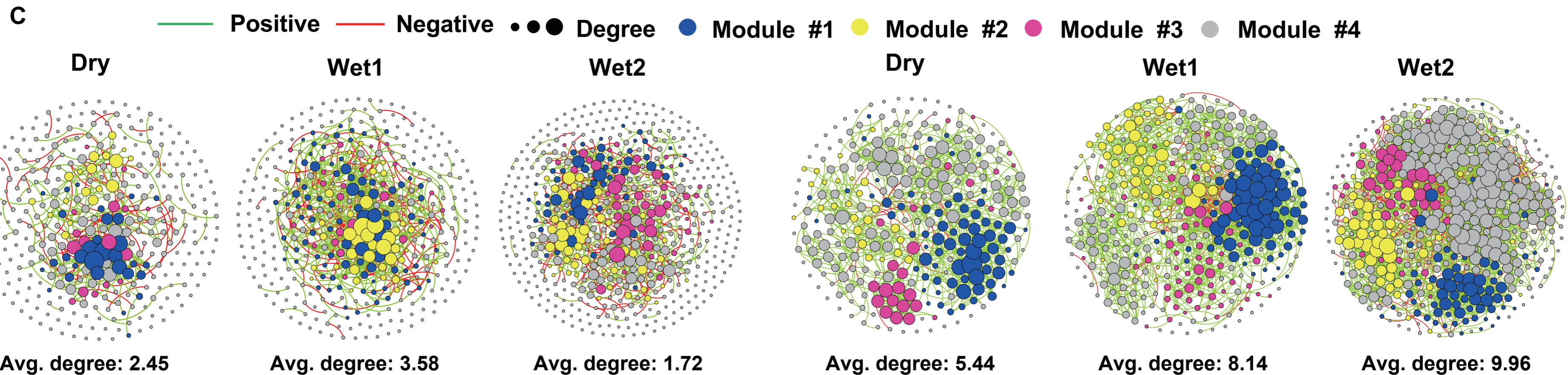

Supplement: Supplementary file 1 [file jof-08-00948-s001.zip › Supplementary materials/Figure S5.pdf]
